# Bioinformatics Analysis Identifies Potential Ferroptosis Key Genes in the Pathogenesis of Pulmonary Fibrosis

**DOI:** 10.3389/fgene.2021.788417

**Published:** 2022-01-06

**Authors:** Jie He, Xiaoyan Li, Mi Yu

**Affiliations:** ^1^ Clinical Medical College of Chengdu Medical College, Chengdu, China; ^2^ Department of Pulmonary and Critical Care Medicine, The First Affiliated Hospital of Chengdu Medical College, Chengdu, China; ^3^ Department of Endocrinology, The First Affiliated Hospital of Chengdu Medical College, Chengdu, China

**Keywords:** ferroptosis, pulmonary fibrosis, bioinformatics analysis, gene expression omnibus, treadmill exercise

## Abstract

**Objective:** Ferroptosis has an important role in developing pulmonary fibrosis. The present project aimed to identify and validate the potential ferroptosis-related genes in pulmonary fibrosis by bioinformatics analyses and experiments.

**Methods:** First, the pulmonary fibrosis tissue sequencing data were obtained from Gene Expression Omnibus (GEO) and FerrDb databases. Bioinformatics methods were used to analyze the differentially expressed genes (DEGs) between the normal control group and the pulmonary fibrosis group and extract ferroptosis-related DEGs. Hub genes were screened by enrichment analysis, protein-protein interaction (PPI) analysis, and random forest algorithm. Finally, mouse pulmonary fibrosis model was made for performing an exercise intervention and the hub genes’ expression was verified through qRT-PCR.

**Results:** 13 up-regulated genes and 7 down-regulated genes were identified as ferroptosis-related DEGs by comparing 103 lung tissues with idiopathic pulmonary fibrosis (IPF) and 103 normal lung tissues. PPI results indicated the interactions among these ferroptosis-related genes. Kyoto Encyclopedia of Genes and Genomes (KEGG) Pathway enrichment and Genome-Ontology (GO) enrichment analyses showed that these ferroptosis-related genes involved in the organic anion transport, response to hypoxia, response to decrease oxygen level, HIF-1 signaling pathway, renal cell carcinoma, and arachidonic acid metabolism signaling pathway. The confirmed genes using PPI analysis and random forest algorithm included *CAV1, NOS2, GDF15, HNF4A, and CDKN2A*. qRT-PCR of the fibrotic lung tissues from the mouse model showed that the mRNA levels of *NOS2* and *GDF15* were up-regulated, while *CAV1* and *CDKN2A* were down-regulated. Also, treadmill training led to an increased expression of *CAV1* and *CDKN2A* and a decrease in the expression of *NOS2* and *GDF15*.

**Conclusion:** Using bioinformatics analysis, 20 potential genes were identified to be associated with ferroptosis in pulmonary fibrosis. *CAV1, NOS2, GDF15*, and *CDKN2A* were demonstrated to be influencing the development of pulmonary fibrosis by regulating ferroptosis. These findings suggested that, as an aerobic exercise treatment, treadmill training reduced ferroptosis in the pulmonary fibrosis tissues, and thus, reduces inflammation in the lungs. Aerobic exercise training initiate concomitantly with induction of pulmonary fibrosis reduces ferroptosis in lung. These results may develop our knowledge about pulmonary fibrosis and may contribute to its treatment.

## Introduction

Idiopathic pulmonary fibrosis (IPF) is known as a progressive, chronic, and irreversible lung disease that has symptoms such as the irreversible decline in lung function, progressive pulmonary scarring, and common interstitial pneumonia (Ding et al., 2015; Fanny et al., 2018; Kamio et al., 2018). IPF has effects on more than 3 million people worldwide. The global annual incidence of IPF is 2- 9 people per 100, 000 (Hutchinson et al., 2015) and the median survival time is solely 2–3 years after diagnosis is achieved (Ohta et al., 2017). Previous studies have shown that the risk factors of IPF include genetic factors, smoking, airway inflammation, occupational exposure, etc(H. Y. Lee et al., 2020). Basically, it is currently accepted that the development and progression of pulmonary fibrosis are attributable to aberrant repair following repeated alveolar epithelial cell injury in response to different stimuli. However, its exact pathogenesis is not clear yet (Wolters et al., 2018; Wu et al., 2020).

Recently, an iron-dependent and non-apoptotic regulatory cell death mechanism have been discovered and named ferroptosis (Zhang et al., 2021). Ferroptosis is widely involved in the development of stroke, brain injury, and tumors (Bu et al., 2021; Guan et al., 2021; Hu et al., 2021). However, iron is an essential reagent for normal physiological activities of cells, ferroptosis is distinguished from other forms of cell death such as apoptosis, necrosis, and autophagy by the lethal iron-dependent accumulation of lipid-based reactive oxygen species (ROS) (Ye et al., 2019). The reason is that the excessive accumulation of iron in cells causes lipid peroxidation elevation leading to this type of cell death (Nishizawa et al., 2021). Notably, ferroptosis-related heterozygous gene GPX4 flawed mice showed more severe bleomycin-induced pulmonary fibrosis, while this effect was attenuated in transgenic mice (Tsubouchi et al., 2019), indicating that ferroptosis may be substantially contributing to the pulmonary fibrosis. In addition, Yatmark et al. (2015) showed that excessive iron load in the lung would lead to pulmonary fibrosis and alveolar epithelial cell damage, and a ferrous ion chelator such as deferoxamine (DFO) can reduce the degree of excessive iron-induced lung injury. Therefore, further investigations are required to detect the ferroptosis-related genes including those involved in the pulmonary fibrosis that is largely unknown at present. Since probing and discovering the underlying mechanism of ferroptosis involvement in pulmonary fibrosis can be a potential therapeutical target for its treatment.

Currently, to the knowledge of authors, no bioinformatic-based study has targeted the mechanism underlying ferroptosis genes’ contribution to pulmonary fibrosis. Therefore, in the present study, data analysis and data mining techniques were used to screen the differentially expressed genes (DEGs) in IPF patients compared to the normal lung tissues. Then, the identified DEGs were intersected with the ferroptosis data set, and the random forest algorithm was used to achieve the key ferroptosis DEGs. We also created mice models of pulmonary fibrosis using bleomycin-induced mice and tested these hypotheses through a treadmill training intervention. Our results will contribute to the understanding of the ferroptosis mechanism in pulmonary fibrosis as well as providing new ideas for IFP clinical diagnosis and its treatment.

## Materials and Methods

Ethics Committee of the First Affiliated Hospital of Chengdu Medical College approved all procedures (Approval No. 2021CYFYIRB-BA-32-01). The animal experiments involved in the research content and process of the project met the ethical requirements of experimental animal welfare and complied with the Animal Protection Law and relevant regulations. The environmental conditions of animal laboratory facilities were in line with the Chinese national standard “Experimental Animals and Environmental Facilities” (GB14925-2010), and the animal feeding management and animal experiment operation were in line with the requirements of the regulations of Chengdu Medical College on the management of experimental animals.

### Animals and Experimental Groups

Twenty-four C57BL/6 mice weighing 20–25 g were purchased from the Animal Experimental Center of Guangxi Medical University. Then, they were randomly divided into four groups (each group including six mice) of control group (Co), exercise group (Exe), bleomycin group (Bleo), and bleomycin + exercise group (Bleo + Exe).

### Animal Model of Bleomycin-Induced Pulmonary Fibrosis

Sulfate of bleomycin (1.5 UI/kg; Meizler Biopharma, SP, Brazil) was administered orotracheally under anesthesia (ketamine 100 mg/kg and xylazine 10 mg/kg) on day 1 of the experimental procedures, which corresponded to first day after the initial physical test (after the 3 days of adaptation). Bleomycin-induced pulmonary fibrosis, when administered intratracheally at doses ranging between 1.25 UI/kg and 4 UI/kg, keeps up the best experimental model available now (Mouratis and Aidinis, 2011).

### Treadmill Exercise Tests and Training

As described in the methodology of the previous studies (Yan et al., 2019; Zhao et al., 2020), this experiment contains treadmill adaptation, tests, and training. Briefly, mice were adapted to a treadmill (15 min/day, 25-degree tilt, 0.2 km/h) for 3 days. Then, they underwent a physical test with the starting speed at 0.2 km/h that was increased by 0.1 km/h every 2.5 min. The test ended by exhaustion, that is when the animal could not run anymore even after 10 mechanical stimuli. Thereafter, animals were included in a 4-week, 60-min exercise training program five times a week, reaching 60 percent of the maximum speed achieved in initial physical tests. Euthanasia was carried out 24 h after the last exercise session. The animals were euthanized according to acceptable method of euthanasia as defined by the American Veterinary Medical Association (AVMA) Guidelines on Euthanasia-Approved Euthanasia Method,2013. Once the animals receive the last exercise session, the animals were euthanized with ketamine/xylazine 100/10 mg/kg body weight intraperitoneally as well as a secondary method of cervical dislocation. At the termination of the experiment, lung tissue was collected for the next experiment after euthanized with ketamine/xylazine 100/10 mg/kg body weight intraperitoneally.

### Next Generation Data Sequencing in GEO

The clinical information for IPF patients was obtained from the Gene Expression Omnibus (GEO) database. We downloaded the dataset GSE150910 (Furusawa et al., 2020) stored by Furusawa from GEO. This dataset has the largest number of samples, covering 103 IPF lung tissues and 103 normal lung tissues. The patients’ information of the GSE150910 dataset used for further analysis and mining is shown in Table 1. Since this dataset was from a public database, patient consent and ethics committee approval were not required.

**TABLE 1:** Basic information of the patients in GSE150910.

	**IPF(N = 103)**	**Control (N = 103)**
Age	60.3 ± 18.3	59.9 ± 10.2
Sex	N = 103	N = 103
Male	57 (55%)	45 (44%)
Female	46 (45%)	58 (56%)
Race	N = 101	N = 103
Non-Hispanic white	85 (84%)	87 (84%)
Hispanic	7 (7%)	4 (4%)
Asian	2 (2%)	3 (3%)
Black	4 (4%)	9 (9%)
Other	3 (3%)	0 (0%)
**Smoke**	N = 95	N = 96
Ever	40 (42%)	43 (45%)
Never	55 (58%)	53 (55%)
Sampling method		
Surgical lung biopsy	36 (35%)	41 (40%)
Transplant	67 (65%)	62 (60%)

### Differential Gene Analysis

For RNA-seq initial data in GSE150910, the Reads count data is standardized by the transcripts per million (TPM). The TPM normalization formula was as follows:

TPM = Read count × 1,000,000/Mapped Reads (Jiao et al., 2018).

The “LIMMA” package in R was used to identify the DEGs. For this purpose, after adjustment, the gene accord with P ＜ 0.05 as well as log_2_Flod change absolute value ＞ 1 were considered as DEGs. Then, a dataset of 259 ferroptosis-related genes was obtained from the ferroptosis database (FerrDb; Zhounan.org; dataset: drivers, suppressor, markers) which were cross-referenced with GSE150910 to recognize DEGs associated with ferroptosis. R software “VennDiagram”, “heatmap” and “ggplot2″ software package were used to plot the Venn diagrams and heat maps. The heatmap was created using Euclidean distance and the Ward method of hierarchical clustering. Furthermore, DEGs associated with ferroptosis were validated using “edgeR” package (log_2_Flod change absolute value ＞ 1, false discovery rate <0.05).

### Analyzing the Ferroptosis-Related Genes by GO and KEGG Pathway Enrichment

“GO Plot” software package in R was used to apply the GO and KEGG pathway enrichment analyses. GO analysis included biological processes (BP), cellular components, and molecular functions (MF) (CC).

### PPI Analysis and Correlation Analysis of Ferroptosis-Related DEGs

The STRING online database (https://string-db.org/) and Cytoscape software (version 3.8.1) were used to analyze the interaction among ferroptosis-related DEGs. The network was set to the cutoff (interaction score >0.15) in the STRING online database. Genes were represented by nodes, and the interactions between the genes were indicated by edges the down-regulated genes were indicated by green and the up-regulated ones were indicated by blue. MCODE is Cytoscape’s application program that was used for gene networks cluster analysis to map the key modules. The genes in the key modules were further screened by a random forest algorithm. The “Corrplot” package in R (a spearman correlation analysis function) was used to recognize the correlation between differentially expressed ferroptosis-related genes. Differences with *p* < 0.05 were considered statistically significant.

### Random Forest Sequencing

An effective and popular classification and regression method for various prediction problems in biological research is the random forest algorithm. For ranking the important indicators, the mean decrease Gini (MDG) in random forest algorithm was applied to quantify which index contributes most to the classification accuracy. MDG is an important associated index as its higher amount illustrates that the degree of category-derived impurity can be reduced farthest by one variable. Here, we used GSE150910 to develop the random forest model with the specific parameters of max features: auto, n estimators: 500 min, sample leaf: 1, and the number of variables tried at each split: 2. The top 5 screened-out hub genes were used for subsequent research and experimental verification.

### qRT-PCR Analysis

After 4 weeks of exercise training (Co: n = 6, Exe: N = 6, Bleo: n = 6, Bleo + Exe: n = 6), the lung tissue of each mouse was extracted to undergo qRT-PCR for validating ferroptosis marker Prostaglandin-Endoperoxide synthase 2(*PTGS2*) and hub genes. In short, the total RNA of each lung tissue sample was collected using TRIzol. The collected RNA purity was determined by the Quantus fluorometer. For this purpose, first, a reverse transcription reaction was performed on the total RNA. The amplified cDNA samples were then mixed with a one-step SYBR PrimeScript PLUS RTPCR kit. Finally, the reaction was performed on AriaMx HRM. The positive control for this reaction was GAPDH and each sample was calculated using the comparative Ct method (Table 2).

**TABLE 2:** Specific primer sequences used in PCR.

Gene		Primer sequences (5′-3′)
CAV1		F:GCAGAACCAGAAGGGACACACAG
		R:ATAGACACGGCTGATGCACTGAATC
NOS2		F:ATCTTGGAGCGAGTTGTGGATTGTC
		R:CTGGGAGGAGCTGATGGAGTAGTAG
GDF15		F:CGCTGCTGTCACTTGGAGACTG
		R:CACCACTGTCTGTCCTGTGCATAAG
HNF4A		F:GCAAGTGAGCCTGGAGGATTACATC
		R:CATCTGTCCATTGCTGAGGTGAGAG
CDKN2A		F:TTCAGGTGATGATGATGGGCAACG
		R:CCACCCAGCGGAACACAAAGAG
PTGS2		F:CTGGTGCCTGGTCTGATGATGTATG
		R:GGGTGCCAGTGATAGAGTGTGTTG
GAPDH		F:CAGCCGCATCTTCTTGTGC
		R:GGTAACCAGGCGTCCGATA

### Survival Analysis of the Hub Genes

We downloaded the GSE70866 dataset (Prasse et al., 2019) from the GEO database. This dataset contains gene expression profile data and survival prognostic information from 212 patients with IPF. We used this dataset to validate the survival prognosis of the hub genes. The Kaplan-Meier method was applied to analyze this small sample size. The survival software package in R was used for the survival analysis. For analyzing the difference in the survival curves of IPF patients with different levels of hub gene expression, the log-rank test provided in the survival package was used.

### Statistical Analysis

For plotting and performing the statistical analysis, we used GraphPad Prism 8.0 and R (3.6.1) software. All data are expressed as mean ± standard deviation (SD). For sequencing data, the “LIMMA” package in R was used for differential gene analysis, and the “randomForest” package in R was used for hub gene sequencing. ANOVA was used for statistical analysis of the control group (Co), exercise group (Exe), and the bleomycin + exercise group (Bleo + Exe), followed by Tukey multiple comparison post-mortem tests. *t*-test was applied for determining the *p*-value in the differentially expressed genes and the adjusted *p*-value, where the *p*-value was adjusted by False Discovery Rate (FDR). Differences with *p* < 0.05 were considered statistically significant.

## Results

### Differential Expression of Ferroptosis-Related DEGs

**FIGURE 1:**
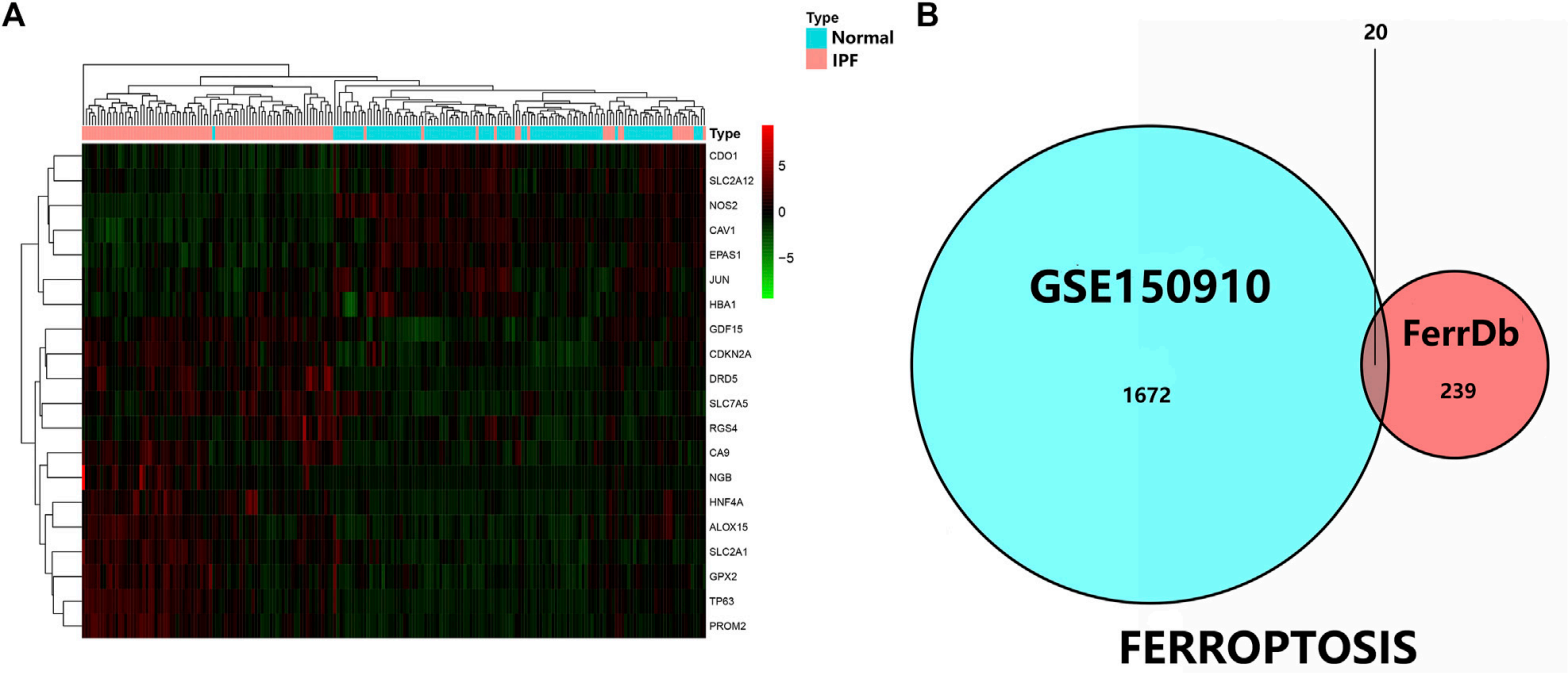
**(A)** Three were 1,692 differentially expressed genes in IPF tissues and normal lung tissues. The first 20 differentially expressed genes were shown in the heatmap, with red representing significantly up-regulated genes and green representing significantly down-regulated genes in the samples. **(B)** Venn diagram of ferroptosis differentially expressed genes. We intersected ferroptosis datasets with GSE150910 to identify ferroptosis differentially expressed genes.

As was previously mentioned, the dataset GSE150910 was downloaded for IPF next generation sequencing (NGS) from the GEO database. The expression profile of mRNA was normalized in transcripts per million (TPM). Comparing the absolute log_2_Flod change values of IPF lung tissue and normal lung tissue, the 1,692 differentially expressed genes (DEGs) were obtained. For identifying the ferroptosis-related DEGs, a dataset of 259 genes was obtained from the ferroptosis Data (FerrDb) which was then crossed with GSE150910 dataset. As the result, seven down-regulated DEGs and thirteen up-regulated DEGs were detected (Table 3). Figure 1 illustrates the heat map and Venn diagram of DEGs. Then, DEGs were further classified using the FERDB online tool into three categories of ferroptosis drivers (*CA9*, *EPAS1*, *CDO1*, *CDKN2A*, *ALOX15*), ferroptosis suppressors (*TP63*, *CAV1*, *PROM2*, *JUN*), and ferroptosis markers (*NOS2*, *HNF4A*, *RGS4*, *SLC2A1*, *GDF15*, *SLC2A12*, *NGB*, *DRD5*, *GPX2*, *HBA1*). The edgeR results showed that there were 1854 differentially expressed genes between IPF lung tissue and normal lung tissue (Supplementary Table S1). The expression trend of ferroptosis-related DEGs was the same as that of “LIMMA” test.

**TABLE 3:** Ferroptosis differentially expressed genes of IPF.

Gene	log_2_FC	*p*-value	FDR	Gene title
Downregulated genes				
NOS2	−77	3.05E-20	1.53E-19	Nitric Oxide synthase 2
HBA1	−1.67	0.00011	0.000110,466	Hemoglobin Subunit Alpha 1
CAV1	−1.40	1.12E-23	1.12E-22	Caveolin 1
EPAS1	−1.29	4.83E-14	9.66E-14	Endothelial PAS Domain Protein 1
CDO1	−1.23	1.79E-13	2.99E-13	Cysteine dioxygenase Type 1
JUN	−1.13	1.48E-10	1.97E-10	Jun Proto-Oncogene, AP-1 Transcription Factor Subunit
SLC2A12	−1.11	5.51E-16	1.38E-15	Solute Carrier Family 2 Member 12
Upregulated genes				
CDKN2A	1.04	8.00E-17	2.67E-16	Cyclin Dependent kinase Inhibitor 2 A
SLC7A5	1.05	3.79E-10	4.74E-10	Solute Carrier Family 7 Member 5
GPX2	1.06	4.27E-11	6.10E-11	Glutathione peroxidase 2
CA9	1.09	7.13E-09	7.92E-09	Carbonic anhydrase 9
SLC2A1	1.20	6.24E-13	9.60E-13	Solute Carrier Family 2 Member 1
RGS4	1.29	1.47E-06	1.55E-06	Regulator Of G Protein Signaling 4
GDF15	1.50	1.39E-16	3.97E-16	Growth Differentiation Factor 15
ALOX15	1.52	5.11E-10	6.01E-10	Arachidonate 15-Lipoxygenase
HNF4A	1.64	1.93E-18	7.72E-18	Hepatocyte Nuclear Factor 4 Alpha
PROM2	2.24	2.80E-20	1.53E-19	Prominin 2
TP63	2.26	2.23E-15	4.95E-15	Tumor Protein P63
DRD5	2.80	2.86E-26	5.73E-25	Dopamine Receptor D5
NGB	3.61	7.58E-14	1.38E-13	Neuroglobin

FDR: false discovery rate.

### Enrichment Pathways and Analysis of Ferroptosis-Related DEGs

**FIGURE 2:**
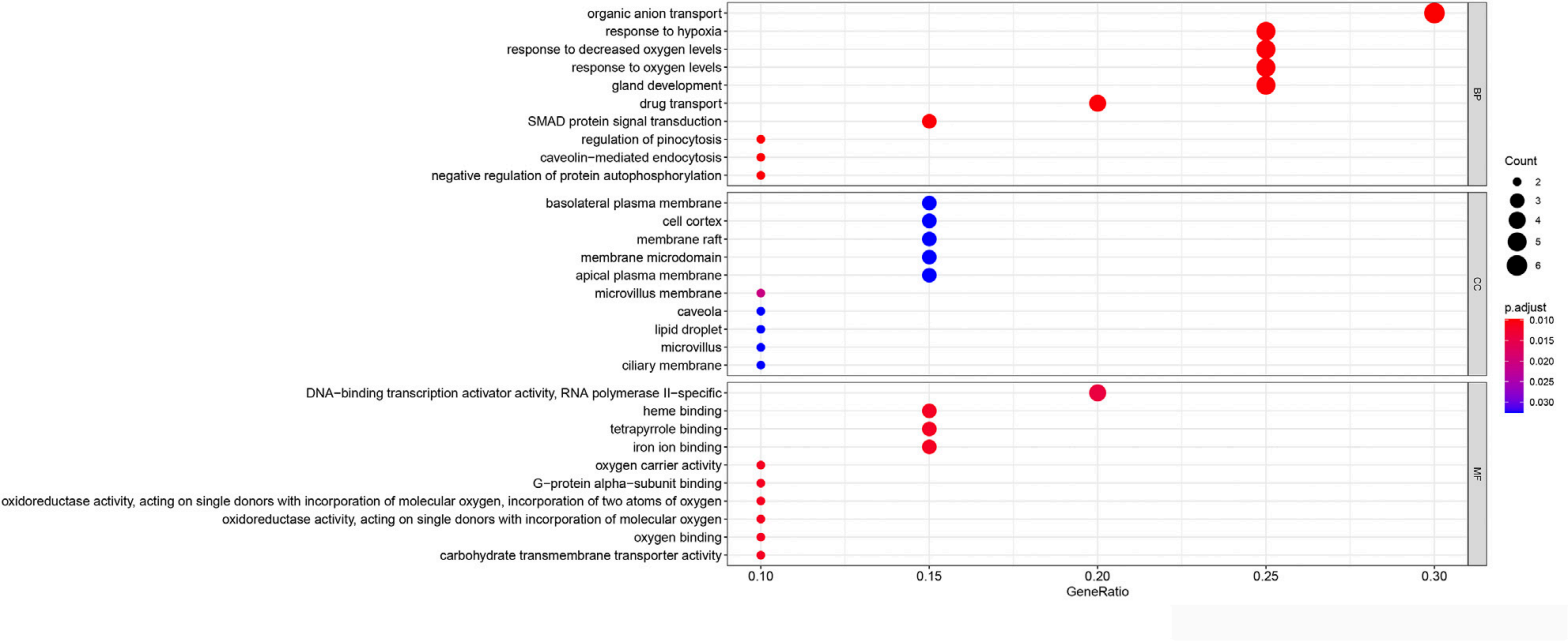
Gene Ontology (GO) enrichment analysis of 20 differentially expressed ferroptosis-related genes. Abbreviation: BP, biological process; CC, cellular component; MF, molecular function.

**FIGURE 3:**
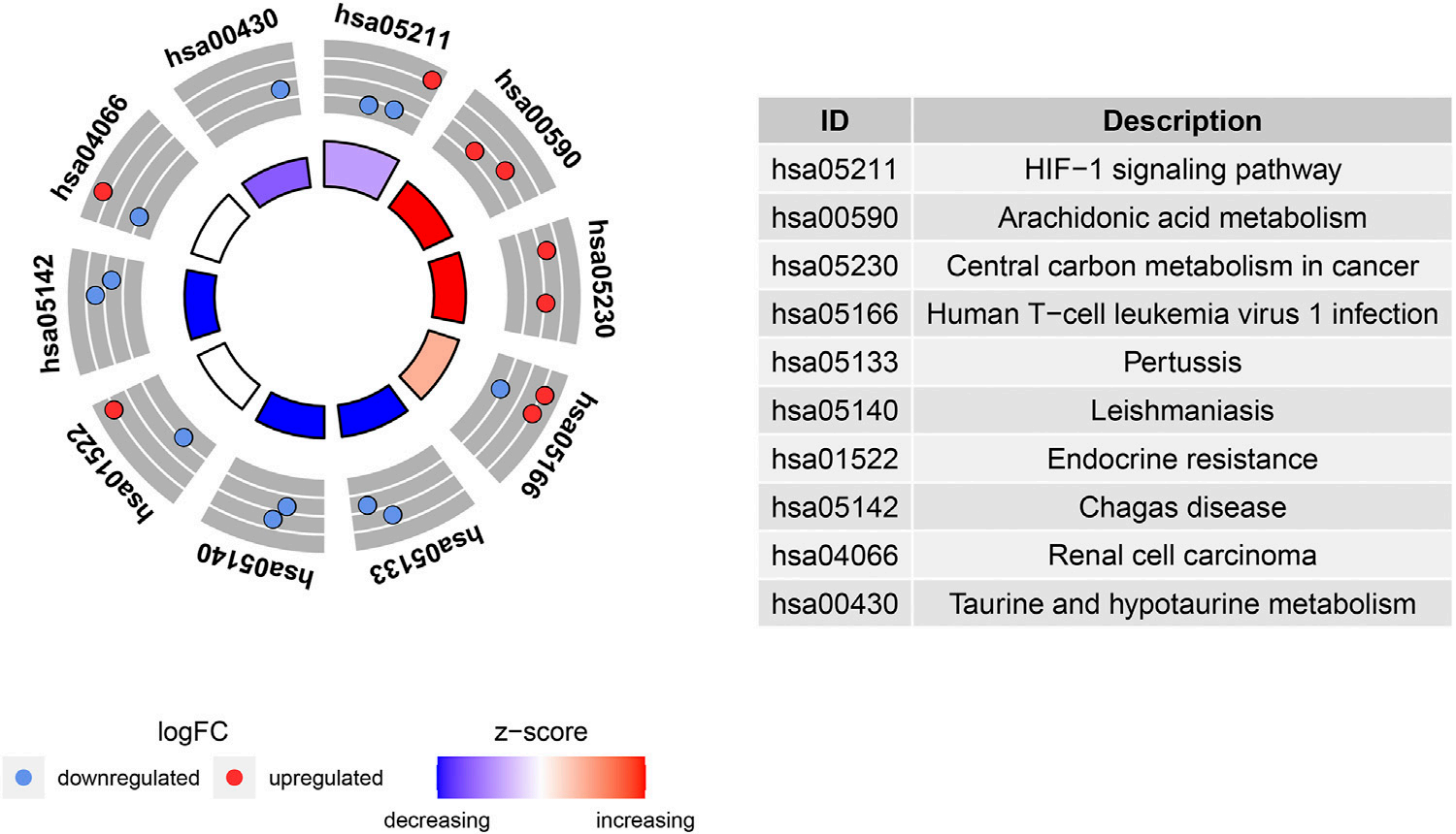
Kyoto Encyclopedia of Genes and Genomes (KEGG) analysis of 20 differentially expressed ferroptosis-related genes.

The potential biological functions of the identified ferroptosis-related DEGs were analyzed using GO and KEGG enrichment analyses by R. Accordingly, the most significant enrichment terms of GO included organic anion transport, response to hypoxia, response to decreased oxygen levels (biological process); basolateral plasma membrane, cell cortex, membrane raft (cellular component); DNA−binding transcription activator activity, RNA polymerase II−specific, heme binding, tetrapyrrole binding (molecular function) (Figure 2). The results of KEGG enrichment analysis showed that ferroptosis-related DEGs were majorly involved in Renal cell carcinoma, Arachidonic acid metabolism, Central carbon metabolism in cancer, Human T−cell leukemia virus 1 infection, Pertussis, Leishmaniasis, Endocrine resistance, Chagas disease, HIF−1 signaling pathway, Taurine and hypotaurine metabolism (Figure 3).

### PPI Network and Correlation Analysis of Ferroptosis-Related DEGs

**FIGURE 4:**
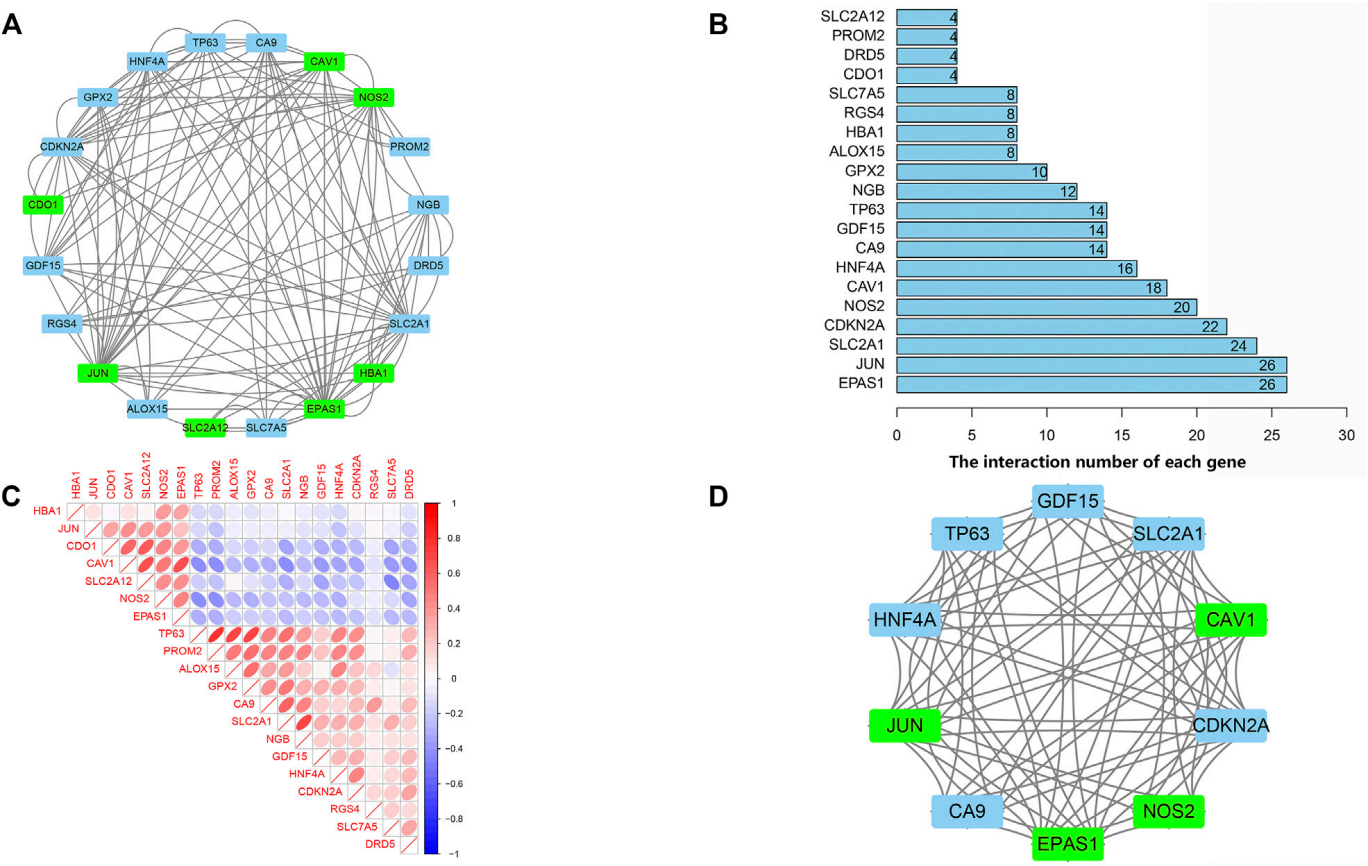
protein-protein interaction (PPI) analysis the 20 differentially expressed ferroptosis-related genes. **(A)** The PPI among 20 differentially expressed ferroptosis-related genes. **(B)** the interaction number of each differentially expressed ferroptosis-related genes. **(C)** Spearman correlation analysis of the 20 differentially expressed ferroptosis-related genes. **(D)** The key module were identified by MCODE, which was used to identify network gene clustering. Green represents downregulated genes, and blue represents upregulated genes.

PPI analyses were performed to determine the interaction between differentially expressed ferroptosis-related genes. According to the results, we found that there were interactions between these ferroptosis-related genes (Figure 4A). Figure 4B shows the number of interactions for each gene. Spearman correlation analysis was used to investigate the correlation of expression in these genes. The findings from the GSE150910 dataset suggested that there was an interaction between 20 differentially expressed ferroptosis-related genes (Figure 4C). Moreover, Cytoscape’s application program MCODE was applied for the cluster analysis of gene networks. As the result, 4 cut genes (*CAV1, JUN, NOS2,* and *EPAS1*) and 6 raised genes (*GDF15, SLC2A1, CDKN2A, CA9, HNF4A*, and *TP63*) were established by drawing key modules (Figure 4D).

### Identifying the Key DEGs Using randomForest Classifier

**FIGURE 5:**
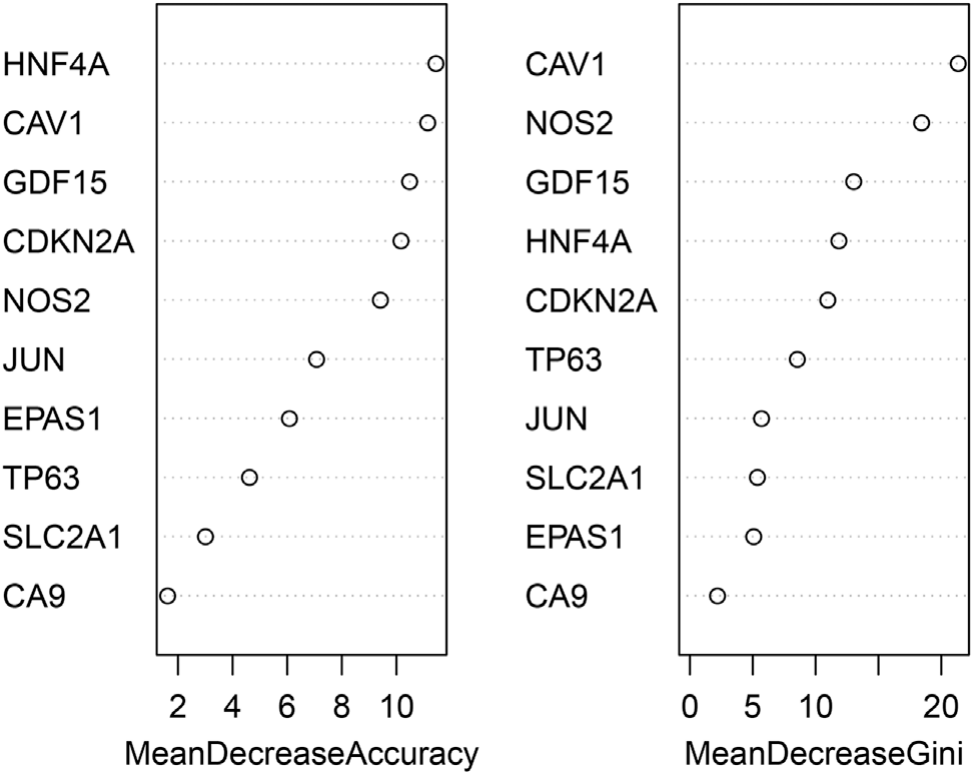
identification of the most important hub genes by using a random forest classifier.

The randomForest classifier (as a package in R software) was used to efficiently predict whether a variable is noise and evaluate its importance. The Out-of-bag (OOB) error of the randomForest model is 7.28%. Mean decrease gini using random forest algorithm to rank the 10 genes that screened by MCODE, and select the top five DEGs (*CAV1, NOS2, GDF15*, *HNF4A, CDKN2A*) for further analysis and validation by animal experiments (Figure 5).

### Potential Biomarker Expression Detected by qRT-PCR

**FIGURE 6:**
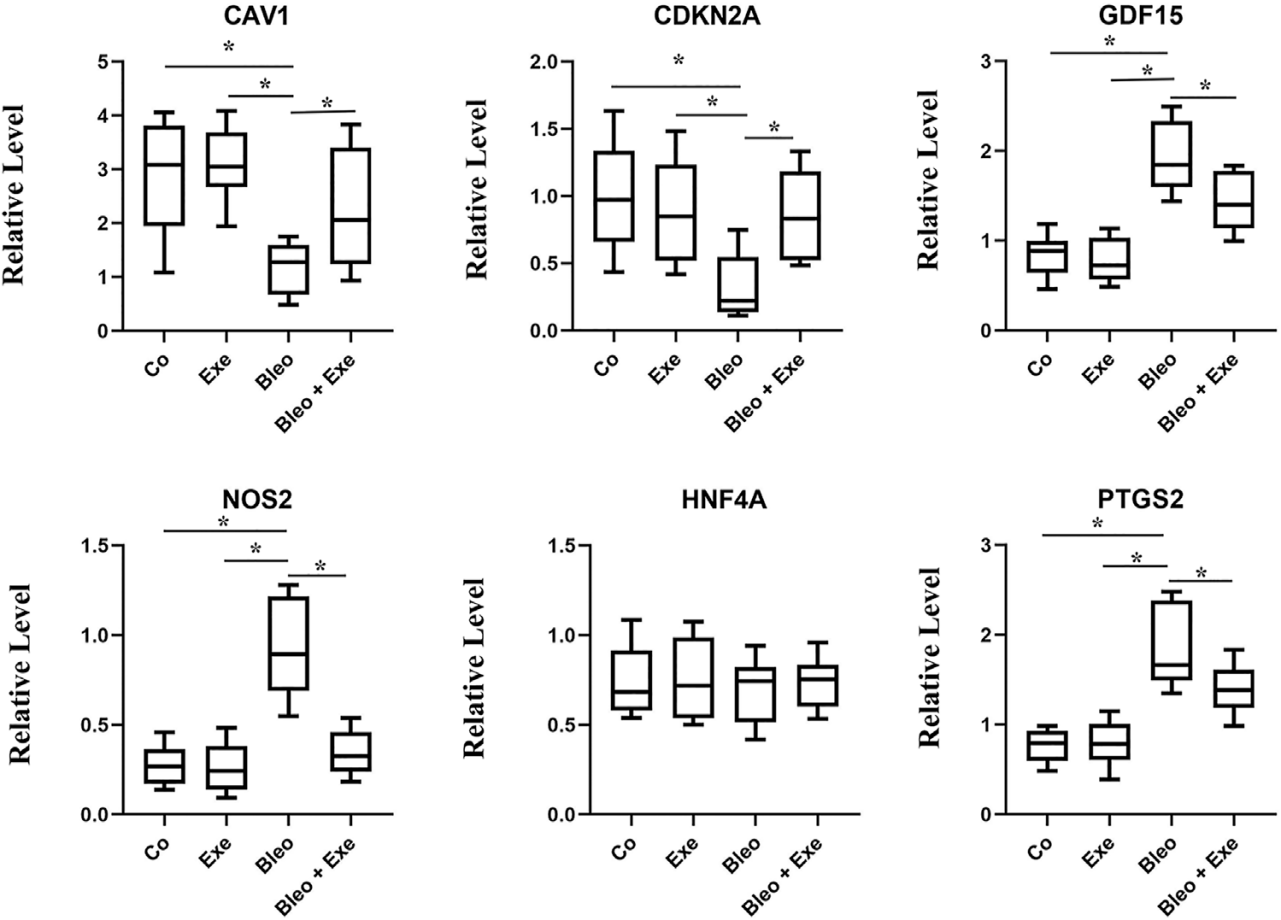
qRT-PCR results show that the expression levels of CAV1 and CDKN2A were obviously lower, and NOS2, GDF15, PTGS2 was higher in pulmonary fibrosis mice than that of healthy controls (all *p* < 0.05). After treadmill training, CAV1 and CDKN2A were obviously upregulated, and NOS2, GDF15 and PTGS2 were downregulated by comparing the treatment group and the model group (all *p* < 0.05). No significant difference was observed in the expression level of HNF4A among the four groups.

The screened biomarkers of pulmonary fibrotic mice models (*CAV1, NOS2, GDF15*, *HNF4A, CDKN2A*) and ferroptosis marker *PDGS2* were verified using qRT-PCR. The results indicated a significant down-regulation in the expression of *CAV1* and *CDKN2A* in IPF tissues compared to the control group, while the expression of *PDGS2*, *NOS2* and *GDF15* was up-regulated in IPF tissues compared to the control group. No significant difference was observed in the expression level of *HNF4A* among the four groups. After exercise training, the expression of *CAV1* and *CDKN2A* increased in the bleomycin + exercise group compared with the bleomycin group, while the expression of *PDGS2*, *GDF15* and *NOS2* had decreased (Figure 6).

### Survival Analysis of Hub Genes in the Validation Group

**FIGURE 7:**
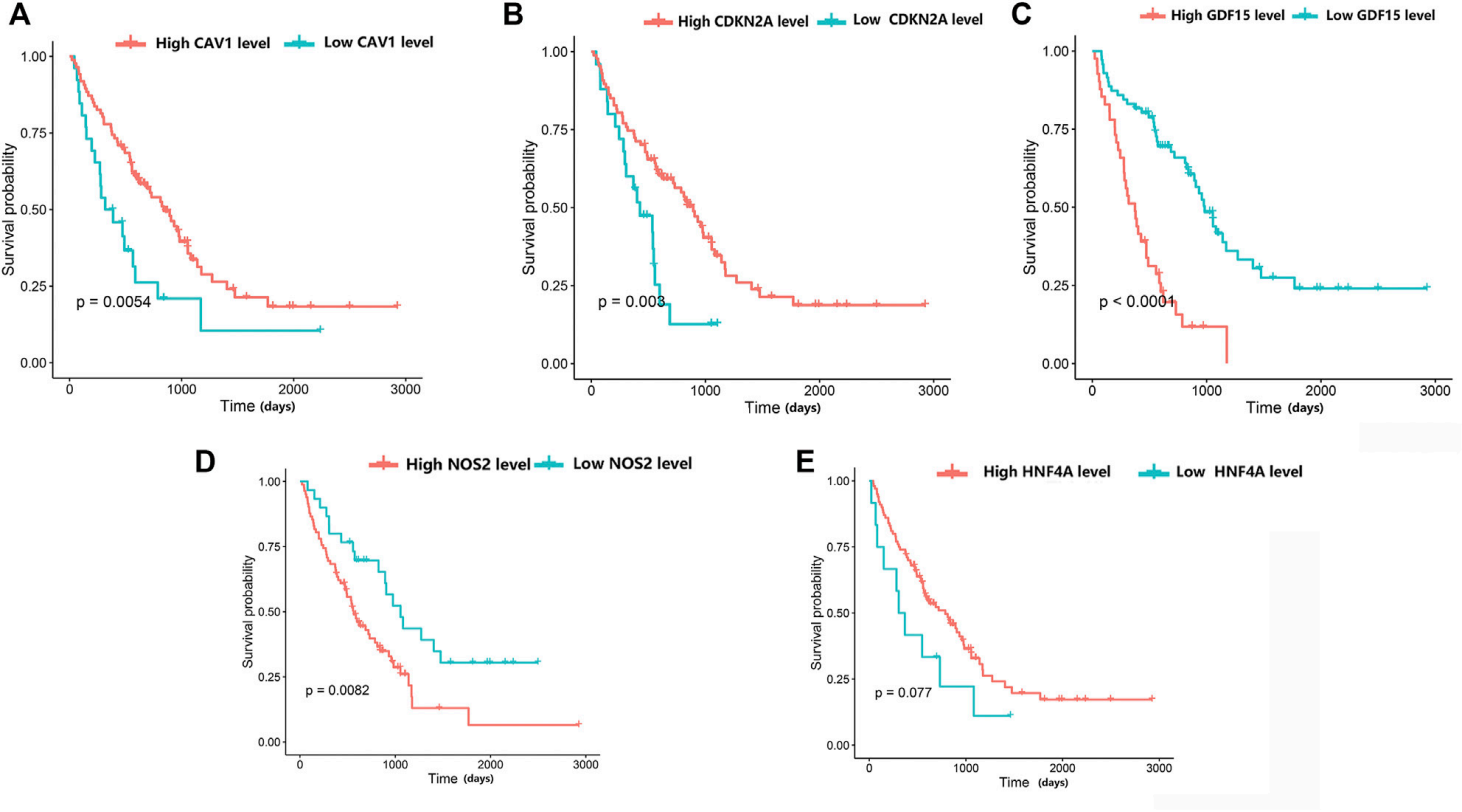
The relationship between hub differentially expressed ferroptosis-related genes and IPF prognosis was evaluated by Kaplan-Meier survival analysis. **(A)** Association between CAV1 expression and survival time in patients with IPF. **(B)** Association between CDKN2A expression and survival time in patients with IPF. **(C)** Association between GDF15 expression and survival time in patients with IPF. **(D)** Association between NOS2 expression and survival time in patients with IPF. **(E)** Association between HNF4A expression and survival time in patients with IPF.

The prognostic values of the five genes diagnosed as the hub in IPF patients were investigated by the overall survival (OS) analysis using the GSE70866 dataset. The results indicated a poorer overall survival in IPF patients with higher expression levels of *NOS2* and *GDF15* compared to patients with lower expression levels for these two genes (*p* < 0.05). Additionally, the lower expression levels of *CAV1* and *CDKN2A* in IPF patients were associated with poorer overall survival compared to patients with high expression levels. There was no significant correlation between the expression level of *HNF4A* and the IPF patients’ OS (Figure 7).

## Discussion

The present study identified the key ferroptosis-related genes in pulmonary fibrosis and explored the potential pathogenesis of ferroptosis in pulmonary fibrosis. Here, a total of 20 DEGs were obtained from the cross set of GSE150910 and FerrDb datasets, including and 13 upregulated genes and 7 downregulated genes with significant interactions among them. Then, the results of enrichment analysis using R software indicated that these genes were mainly involved in the HIF1-α signaling pathway and hypoxia response. Therefore, we used treadmill training to verify the pulmonary fibrosis mice model. Animal experiments showed that exercise might affect the expression of ferroptosis-related genes and inhibit ferroptosis in pulmonary fibrosis confirming the reliability of bioinformatics analysis. Additionally, this project achieved several genes that have not been previously related to ferroptosis and/or pulmonary fibrosis. From a bioinformatics perspective, the present study has provided a substantial reference for the potential mechanisms of pulmonary fibrosis pathogenesis.

In general, the excess accumulation of iron, the lipid-based intracellular ROS overaccumulation, and eventually the lipid oxidation are the main characterizations of ferroptosis which cause damages to the cell membrane leading to cell death (Honarpisheh et al., 2016). This type of cell death often occurs due to the oxidative stress caused by the excess ROS mainly accumulated during the aerobic metabolisms (Ning et al., 2017). Our enrichment analyses showed that after such oxidative stresses, some signaling pathways such as the HIF1-α signaling pathway are activated that can lead to ferroptosis or cell apoptosis through inducing free radicals’ generation after pulmonary fibrosis (Epstein Shochet et al., 2021). The application of antioxidants for treating pulmonary fibrosis has been widely studied (Venkatadri et al., 2017), one reason of which, can be their potential of reducing ferroptosis during pulmonary fibrosis. It has been previously confirmed that iron overaccumulation can activate the HIF1-α signaling pathway. Therefore, it is hypothesized that inhibiting this activation process can reduce the intracellular content of iron and ameliorate the alveolar epithelial cell function (Speer et al., 2013; Xu et al., 2017). In consistence with our bioinformatics analysis, these results suggest that the HIF1-α signaling pathway may be activated by ferroptosis, promote ROS production, and aggravate cell damage, leading to a vicious cycle.

Furthermore, we obtained a module composed of 10 ferroptosis-related genes (*CAV1, JUN, NOS2, EPAS1, GDF15, SLC2A1, CDKN2A, CA9*, *HNF4A, TP63*) using PPI analysis. These genes received a random forests sequencing, and the top five genes were screened as *CAV1, NOS2, GDF15*, *HNF4A, CDKN2A*, respectively. Random forest algorithm is a classifier in machine learning. It has good adaptability to complex data and can sort various variables with high prediction accuracy and improve test efficiency (Liu et al., 2021). Accordingly, our five screened-out ferroptosis-related genes showed high reliability. CAV1 is a membrane-bound scaffold protein family which is related to CAV2 and CAV3 protein families (Lillo Urzúa et al., 2020). CAV1 is widely expressed in lung tissues including alveolar epithelial cells, endothelial cells, fibroblasts, and smooth muscle cells. Therefore, the CAV1 protein loss can cause a variety of respiratory diseases (Royce and Le Saux, 2014; Fang et al., 2017; Li et al., 2019). Many studies have also discussed that CAV1 expression is significantly reduced in lung tissues of IPF patients, which is consistent with the results of our bioinformatics analysis. The reason is that normal expression of CAV1 can prevent TGF-βR (the growth factor-β receptor) from transforming, and thus, inhibits the overexpression of transforming growth factor (TGF-β). Reduced expression of CAV1 will weaken the inhibitory effect of TGF-βR, thereby, activating the TGF-β signaling pathway, resulting in a large amount of extracellular matrix (ECM) generation, causing the occurrence of pulmonary fibrosis (Lee et al., 2007; He et al., 2015; Budi et al., 2016). In our pulmonary fibrosis mice model, it was further demonstrated that the mRNA expression level of *CAV1* in bleomycin-induced pulmonary fibrosis tissues was significantly lower compared to the normal control group. Moreover, the overall survival analysis verified a longer survival time in patients with high *CAV1* expression. In the same regard, Lin et al. (2019). indicated that CAV1 is a major component of cellulose that regulates cell signaling and endocytosis. Overexpression of CAV1 can reduce infiltration of neutrophils and monocytes/macrophages and prevent bleomycin-induced pulmonary fibrosis, which may be related to its key regulatory role in inflammatory activities. Interestingly, our animal experiment showed that *CAV1* mRNA expression increased when treadmill exercise was performed in mice with pulmonary fibrosis. El-Mafarjeh et al. (El-Mafarjeh et al., 2020) showed that treadmill exercise improves pulmonary and systemic inflammation in bleomycin-induced pulmonary fibrosis models. This may be related to the fact in this study that treadmill increases CAV1, but the specific mechanisms need to be confirmed by further experiments. CDKN2A, known as the cycle-dependent kinase inhibitor gene, belongs to the cycle-dependent kinase inhibitor family and is an important tumor suppressor gene (Llanos et al., 2015). Du et al. (2018). showed that the lower expression level of *CDKN2A* mRNA in peripheral blood of IPF patients compared to the control group may be related to the activation of the P53 signaling pathway. In the present experiment, the lower expression level of *CDKN2A* mRNA in the lung tissues of mice with pulmonary fibrosis compared to that of the normal control group was consistent with the results of the a forementioned study. FERDB online tool predicted that *CDKN2A* was associated with ferroptosis and classified it as ferroptosis driver. The hypothesis is based on Chen’s study (Chen et al., 2017). However, Chen et al. did not directly demonstrate that CDKN2A could play a major role in promoting ferroptosis. Whether *CDKN2A* is a driver of ferroptosis in IPF had not been defined. Thus, our results suggested that CDKN2A influencing the ferroptosis of pulmonary fibrosis, the mechanism for which warranted further study. NOS2 is a key gene of oxidative metabolism. Previous studies have reported that under pathophysiological conditions of pulmonary fibrosis, an abnormally high concentration of nitric oxide exists, which may be due to the elevated activity of inducible nitric oxide synthase (NOS2). Excessive nitric oxide production may contribute to fibrosis development (Jang et al., 2004). GDF15 is an endocrine hormone and a member of the transforming growth factor β superfamily that promotes ferroptosis in tumor cells (Shao et al., 2021). Zhang (Zhang et al., 2019) et al. reported that GDF15 is a secreted protein of epithelial origin and may be a useful biomarker of epithelial stress since its increased expression associates with a poorer prognosis in IPF patients, which is consistent with the findings of the present bioinformatics analyses in this study. We speculate that GDF15 may promote ferroptosis in pulmonary fibrosis, thus, aggravate the inflammatory responses in lung tissues and accelerate the process of pulmonary fibrosis. HNF4A is a nuclear transcription factor that binds to DNA as a homodimer and mainly relates to fibrosis development in the liver. Knocking down HNF4A has been shown that can alleviate liver fibrosis (Ji et al., 2019). However, no studies have been reported on *HNF4A* association with pulmonary fibrosis. Similarly, the animal experiments and survival analysis of bioinformatics analysis in this study showed that *HNF4A* has no association with pulmonary fibrosis. Therefore, the relationship between *HNF4A* and pulmonary fibrosis remains unclear and needs further verification by fundamental experiment study. Notably, Data mining showed that *NOS2* and *CAV1* were downregulated, and *CDKN2A* and *GDF15* were upregulated in IPF tissues. However, *NOS2* was upregulated and *CDKN2A* was downregulated in bleomycin-induced mouse IPF tissues. The inconsistency between experimental results and those obtained using bioinformatic analysis may be due to the following reasons. The data used for the bioinformatic analysis was collected on IPF patients, while experimental data derived from bleomycin-induced mice. Although, human and mouse had very high genetic homology, different species may lead to some bias in results. In an addition, some gene expansions might be greatly temporally and spatially specific, which resulted in the discordance of bioinformatic analysis and experimental results. We will collect samples from IPF patients and perform more extensive validation studies in the future.

Nevertheless, our study has some limitations. 1) Obtaining bioinformatics results from lung tissues of IPF patients and normal lung tissues as well as insufficient collection of clinical tissue samples for validation due to experimental conditions and hospital size. 2) Verification of expression levels of different ferroptosis genes in animal models and lack of potential study of hub genes in cellular models of pulmonary fibrosis. Therefore, further verification is required in the future. 3) The mRNA expression values were further standardized by different standardization method, which might yield differing findings. Most of the data to date is restricted to the detection of changes in gene expression and needs to be validated by further functional experiments.

## Conclusion

We identified 20 potential ferroptosis-related genes in pulmonary fibrosis by bioinformatics analysis. *CAV1*, *NOS2*, *GDF15*, *CDKN2A* may influence the development of pulmonary fibrosis by regulating ferroptosis. Aerobic exercise training after induction of pulmonary fibrosis may attenuate ferroptosis in lung. These results may expand our understanding of pulmonary fibrosis and may contribute to the treatment of pulmonary fibrosis.

## Data Availability

The original contributions presented in the study are included in the article/Supplementary Material, further inquiries can be directed to the corresponding author.
